# Neonatal Disruption of Serine Racemase Causes Schizophrenia-Like Behavioral Abnormalities in Adulthood: Clinical Rescue by D-Serine

**DOI:** 10.1371/journal.pone.0062438

**Published:** 2013-04-22

**Authors:** Hiroko Hagiwara, Masaomi Iyo, Kenji Hashimoto

**Affiliations:** 1 Division of Clinical Neuroscience, Chiba University Center for Forensic Mental Health, Chiba, Japan; 2 Department of Psychiatry, Chiba University Graduate School of Medicine, Chiba, Japan; Baylor College of Medicine, United States of America

## Abstract

**Background:**

D-Serine, an endogenous co-agonist of the *N*-methyl-D-aspartate (NMDA) receptor, is synthesized from L-serine by serine racemase (SRR). Given the role of D-serine in both neurodevelopment and the pathophysiology of schizophrenia, we examined whether neonatal disruption of D-serine synthesis by SRR inhibition could induce behavioral abnormalities relevant to schizophrenia, in later life.

**Methodology/Principal Findings:**

Neonatal mice (7–9 days) were injected with vehicle or phenazine methosulfate (Met-Phen: 3 mg/kg/day), an SRR inhibitor. Behavioral evaluations, such as spontaneous locomotion, novel object recognition test (NORT), and prepulse inhibition (PPI) were performed at juvenile (5–6 weeks old) and adult (10–12 weeks old) stages. In addition, we tested the effects of D-serine on PPI deficits in adult mice after neonatal Met-Phen exposure. Finally, we assessed whether D-serine could prevent the onset of schizophrenia-like behavior in these mice. Neonatal Met-Phen treatment reduced D-serine levels in the brain, 24 hours after the final dose. Additionally, this treatment caused behavioral abnormalities relevant to prodromal symptoms in juveniles and to schizophrenia in adults. A single dose of D-serine improved PPI deficits in adult mice. Interestingly, chronic administration of D-serine (900 mg/kg/day from P35 to P70) significantly prevented the onset of PPI deficits after neonatal Met-Phen exposure.

**Conclusions/Significance:**

This study shows that disruption of D-serine synthesis during developmental stages leads to behavioral abnormalities relevant to prodromal symptoms and schizophrenia, in later life. Furthermore, early pharmacological intervention with D-serine may prevent the onset of psychosis in adult.

## Introduction

Accumulating evidence suggests that hypofunction of glutamatergic neurotransmission via the *N*-methyl-D-aspartate (NMDA) receptor plays a crucial role in the pathophysiology of schizophrenia [1]–[6]. D-Serine, an endogenous co-agonist of the NMDA receptor is synthesized from L-serine by serine racemase (SRR) [7]–[9]. Recent studies using *Srr* knock-out (KO) mice show that levels of D-serine in the forebrain of *Srr*-KO mice are 80–90% lower than in wild-type (WT) mice [10]–[13], implying that D-serine production in the forebrain is largely dependent on SRR activity. D-Serine also plays a crucial role in neurotransmission via the NMDA receptor, throughout development and into adulthood [14]–[16].

Several studies have highlighted evidence suggesting that disturbed NMDA receptor neurotransmission due to decreased D-serine levels, is a causative factor in the pathophysiology of schizophrenia [3], [5], [6], [17]–[19]. These findings include firstly, lower levels of D-serine in the blood, cerebrospinal fluid, and postmortem brain tissue from patients with schizophrenia, relative to normal controls [20]–[24]. Secondly, treatment with D-serine is beneficial for reducing positive, negative and cognitive symptoms in patients with schizophrenia [25], [26]. A recent meta-analysis supports findings that D-serine is effective in the treatment of schizophrenia [27]. Thirdly, mRNA expression and activity of D-amino acid oxidase (DAAO), which metabolizes D-serine, is increased in the postmortem brains of schizophrenic patients [28], [29]. Fourthly, the G72 gene at chromosome 13q is significantly associated with schizophrenia [30], [31]. This gene has been designated a DAAO activator, since the G72 protein interacts physically with DAAO [30]. A recent meta-analysis provided evidence of highly significant association between nucleotide variations in the G72/G30 region and schizophrenia [32]. In addition, multiple epidemiological surveys support the neurodevelopmental hypothesis for the pathogenesis of schizophrenia [33]–[35]. Taken together, these findings point to the possibility that hypofunction of the NMDA receptor, resulting from reduced D-serine levels during gestation could interfere with normal fetal brain neurodevelopment and that these deficits are causative to the onset of schizophrenia in adulthood.

Phenazine methosulfate (Met-Phen) is an inhibitor (IC_50_ = 3.0 µM) of SRR [36]. At present, there are no reports of using Met-Phen for *in vivo* manipulation of SRR in brain. Furthermore, the effect of Met-Phen on brain levels of D-serine relative to other amino acids is currently unknown. This study was, therefore, undertaken to examine whether neonatal Met-Phen exposure in mice could lead to behavioral phenotypes similar to those seen in juvenile and adult human schizophrenia. Auditory sensory gating prepulse inhibition (PPI) deficits were used as an animal model of schizophrenia. We also assessed effects of D-serine on behavioral abnormalities in adult mice after neonatal Met-Phen exposure. Finally, we examined whether D-serine could prevent the onset of schizophrenia-like behavior in adult mice after neonatal Met-Phen exposure.

## Materials and Methods

### Animals

DDY mice (Japan SLC Inc., Shizuoka, Japan) were mated at age 10 weeks and the offspring were used for these experiments. Male and female neonatal mice (10 days old, weight 0.4–0.6 g), male juvenile mice (5–6 weeks old, weight 24–30 g) and male adult mice (10–12 weeks old, weight 34–44 g) bred in our laboratory were used for experiments. Animals were housed under controlled temperature and 12 h light/dark cycles (lights on between 07∶00–19∶00), with ad libitum food and water. This study was carried out in strict accordance with the recommendations in the Guide for the Care and Use of Laboratory Animals of the National Institutes of Health, USA. The protocol was approved by the Committee on the Ethics of Animal Experiments of the Chiba University (Permit Number: #22–98). For the measurement of amino acids, mice were sacrificed under CO_2_, and all efforts were made to minimize suffering.

### Neonatal Administration of Met-Phen

On postnatal day 7, baby mice were divided randomly into control (saline treated) or phenazine methosulfate treated groups (Met-Phen, #P9625, Sigma-Aldrich, St Louis, MO) [36]. From P7 to P9, the pups were injected intraperitoneally (i.p.) with Met-Phen (3.0 mg/kg/day) or saline (1.0 ml/kg/day). In a preliminary experiment, we examined the effects of Met-Phen on brain levels of D-serine. The dose (3.0 mg/kg/day for 3 days) of Met-Phen established in this experiment was used for this study. Male mice were separated from their mothers after 3 weeks and mice were caged in separate groups, depending on treatment.

### Measurement of Amino Acids in the Brain

At postnatal (P10), juvenile (P35–P42), and adult (P70–P84) stages, mice were sacrificed, and their brains excised for measurement of amino acids. The cerebellum, frontal cortex, hippocampus and striatum were quickly dissected from whole brain after decapitation. The dissected tissues were weighed and stored at −80°C until assayed. Measurements of D- and L- serine, glutamate, glutamine and glycine levels were carried out using a column-switching high performance liquid chromatography (HPLC) system (Shimadzu Corporation, Kyoto, Japan), as described previously [37]–[39].

### Locomotor Activity in Mice

Both horizontal and rearing activity were monitored by an infrared ray passive sensor system (SCANET-SV10, Melquest Ltd, Toyama, Japan), and activity was integrated every 10 minutes, as previously reported [40]–[42]. Individual mice were placed in activity chambers and allowed 2 hours of free exploration as spontaneous activity.

### Novel Object Recognition Test (NORT)

The NORT was performed as previously reported [43]–[45]. Before testing, mice were habituated in the box for 3 days. During a training session, two objects (differing in shape and color but of similar size) were placed in the box 35.5 cm apart (symmetrically), and each animal was allowed to explore in the box for 5 minutes. The animals were considered to be exploring the object when the head of the animal was both facing and within 2.54 cm of the object or when any part of the body, except for the tail was touching the object. The time that mice spent exploring each object was recorded. After training, mice were immediately returned to their home cages, and the box and objects were cleaned with 75% ethanol, to avoid any possible instinctive odorant cues. Retention tests were carried out at one-day intervals, following the respective training. During the retention test, each mouse was reintroduced into their original test box, and one of the training objects was replaced by a novel object. The mice were then allowed to explore freely for 5 minutes, and the time spent exploring each object was recorded. Throughout the experiments, the objects were counter-balanced, in terms of their physical complexity and emotional neutrality. A preference index, that is, the ratio of time spent exploring either of the two objects (training session) or the novel object (retention test session) over the total time spent exploring both objects, was used.

### Acoustic Startle and Prepulse Inhibition (PPI) Test

The mice were tested for their acoustic startle responses in a startle chamber (SR-LAB, San Diego Instruments, San Diego, CA), using a standard method described previously [41], [46]. Test sessions started after an initial 10-minute acclimation period in the chamber. The mice were subjected to one of six trials: (1) pulse alone, as a 40 ms broadband burst; a pulse (40 ms broadband burst) preceded by 100 ms with a 20 ms prepulse that was (2) 4 dB, (3) 8 dB, (4) 12 dB, or (5) 16 dB over background (65 dB); and (6) background only (no stimulus). Prepulse inhibition (PPI) was expressed as the percentage decrease in amplitude of startle reactivity, caused by a prepulse (% PPI).

To examine effects of D-serine on PPI deficits in adult mice after neonatal Met-Phen exposure, D-serine (900 mg/kg, Sigma-Aldrich, St. Louis, MO) and saline (10 ml/kg) were administered i.p. The drugs and vehicles were injected i.p., 30 minutes before the start of testing.

### Prevention of Met-Phen-induced PPI deficits by D-serine

To examine whether D-serine could prevent the onset of PPI deficits in adult mice after neonatal Met-Phen exposure, D-serine (900 mg/kg/day for 35 days) or vehicle (saline; 10 ml/kg/day for 35 days) were administered i.p. from P35 to P70; this period is thought to represent pre-adolescence to adulthood. One week (P77) after the last dose of D-serine, PPI testing was performed, as described.

### Statistical Analysis

All data are shown as mean ± standard error of the mean (S.E.M.). The results of D-serine levels, locomotion, NORT and social interaction were analyzed by Student’s t-test. PPI data were analyzed by multivariate analysis of variance (MANOVA). Where relevant at individual dB levels, the data were compared by Student’s t-test or one-way ANOVA, followed LSD test. Significance for results was set at *p*<0.05.

## Results

### Effects of Neonatal Met-Phen Treatment on Levels of Amino Acids in the Brain

Treatment with Met-Phen (3.0 mg/kg/day for 3 days from P7–P9) significantly decreased the levels of D-serine in the frontal cortex (t = 2.131, p<0.05) and cerebellum (t = 2.736, p<0.01) at P10 (**Table 1**), suggesting that Met-Phen affects the synthesis of D-serine. Treatment with Met-Phen also significantly decreased levels of L-serine in the frontal cortex, but not cerebellum. Moreover, the levels of glutamate and glutamine in the frontal cortex and cerebellum of Met-Phen treated mice were significantly higher than those of saline-treated mice (**Table 1**). In contrast, levels of glycine remained the same (**Table 1**).

**Table 1:** Levels of amino acids in the frontal cortex and cerebellum 24 hours after the last administration of Met-Phen.

	Glutamate	Glutamine	Glycine	L-Serine	D-Serine
Frontal cortex					
Control	4.729±0.294	3.649±0.373	1.892±0.442	0.746±0.036	0.150±0.005
Met-Phen	5.857±0.314 (124%)*	4.371±0.141 (120%)*	1.463±0.369 (77.3%)	0.585±0.019 (78.4%)***	0.130±0.004 (86.7%)*
Cerebellum					
Control	3.509±0.220	7.118±0.298	2.602±0.153	0.848±0.034	0.154±0.006
Met-Phen	4.375±0.221 (125%)*	9.200±0.423 (129%)**	2.294±0.153 (88.2%)	0.827±0.039 (97.5%)	0.125±0.006 (81.2%)*

Data (nmol/mg tissue) are expressed as the mean ± SEM (Control: n = 9, Met-Phen: n = 21).

Parenthesis is the percentage of control values.

* p<0.05,

** p<0.01,

*** p<0.001 compared to control group (Student’s t test).

Amino acids in the frontal cortex and hippocampus of 4 week old mice did not differ between the two groups, although levels of D-serine and L-serine in the cerebellum of Met-Phen treated mice were significantly higher than those of controls (**Table S1**). In adult mice (10 weeks old), there were no changes in the levels of amino acids in the frontal cortex and striatum between the two groups. Furthermore, levels of all amino acids in the hippocampus of Met-Phen treated mice were significantly lower than those of controls (**Table S2**).

### Effects of Neonatal Met-Phen Treatment on Locomotor Activity

**Figure 1:**
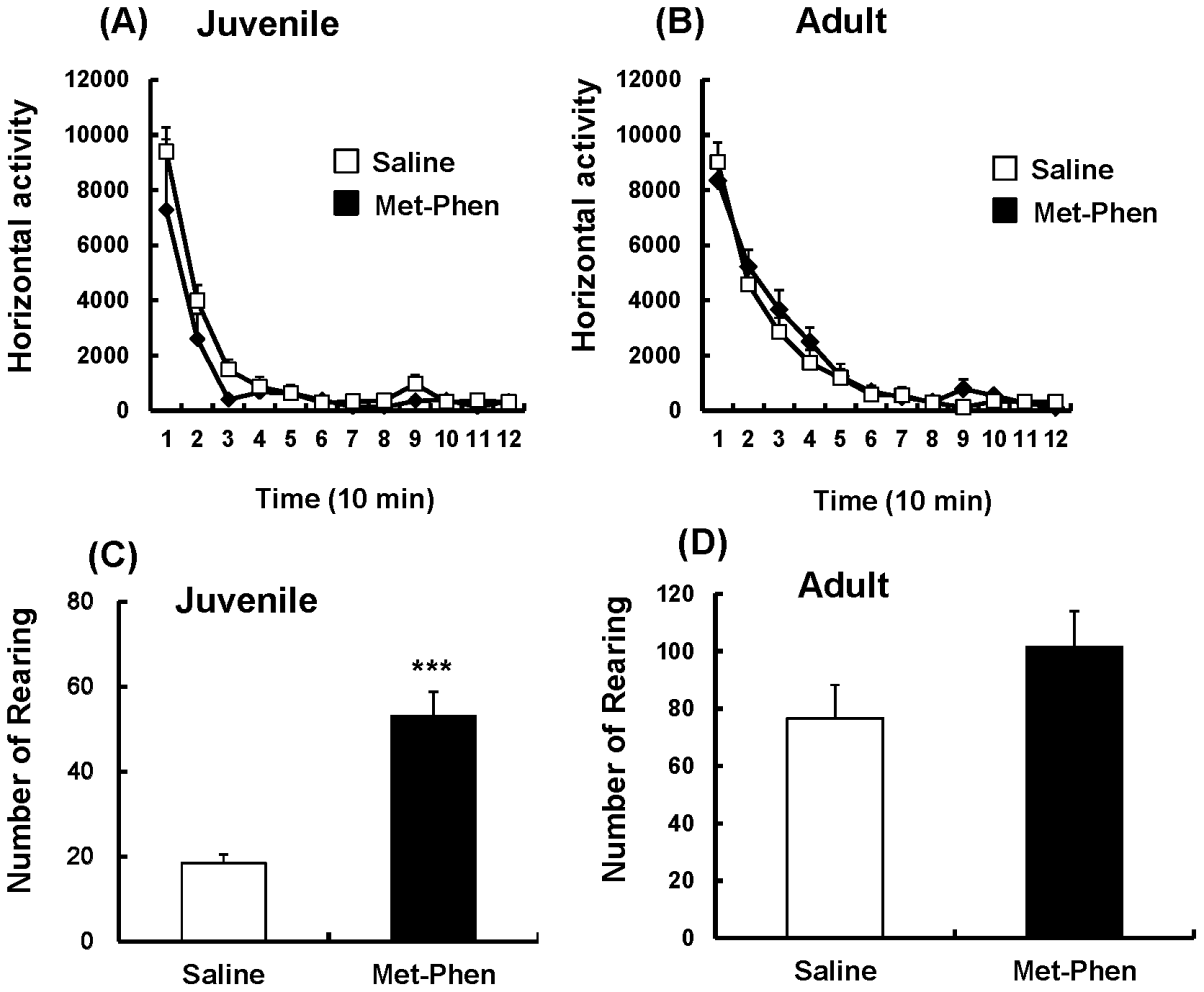
Spontaneous locomotion after neonatal Met-Phen treatment. Saline (1.0 ml/kg/day) or Met-Phen (3.0 mg/kg/day) was administered i.p. from P7 to P9. Horizontal activity and rearing activity were performed at juvenile (5–6 weeks old) and adult stages (10–12 weeks old). Data represent the mean ± S.E.M. (n = 9 mice for control group, n = 16 for Met-Phen group). ***P<0.001 compared with saline treated group.

In the open field test, spontaneous locomotion, such as horizontal activity and rearing activity, was measured at juvenile and adult stages. Horizontal activity was unchanged between the groups at both stages (**Figure 1A and 1B**). However, rearing activity in Met-Phen treated juvenile mice was significantly (t = −5.742, p<0.001) higher than that of the saline treated group (**Figure 1C**), suggesting that Met-Phen treated mice show stereotypy at the juvenile stage. In adult mice, no differences were detected between the treatment groups in rearing activity (**Figure 1D**).

### Effects of Neonatal Met-Phen Treatment on Cognition

**Figure 2:**
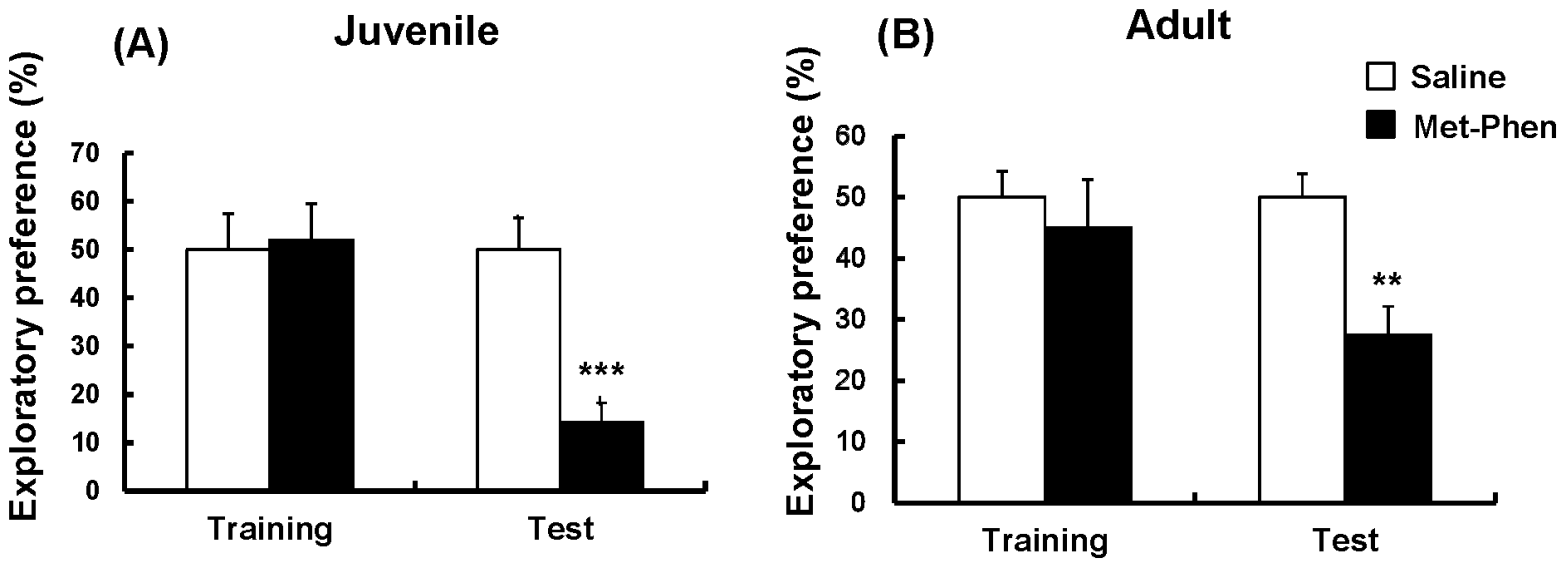
Cognition after neonatal Met-Phen treatment. Saline (1.0 ml/kg/day) or Met-Phen (3.0 mg/kg/day) was administered i.p. from P7 to P9. NORT was performed at juvenile (5–6 weeks old) and adult stages (10–12 weeks old). Data represent the mean ± S.E.M. (n = 8–11 mice for control group, n = 11 or 12 for Met-Phen group). *P<0.05, **P<0.01, *** P<0.001 compared with saline treated group.

Using NORT, we measured cognition at juvenile and adult stages. Treatment with Met-Phen (3.0 mg/kg/day for 3 days from P7–P9) caused significant cognitive deficits in juvenile (t = 4.719, p<0.001) and adult mice (t = 3.458, p<0.01) (**Figure 2A and 2B**). These results imply that neonatal Met-Phen exposure induces cognitive deficits in juvenile and adult mice.

### Effects of Neonatal Met-Phen Treatment on PPI

**Figure 3:**
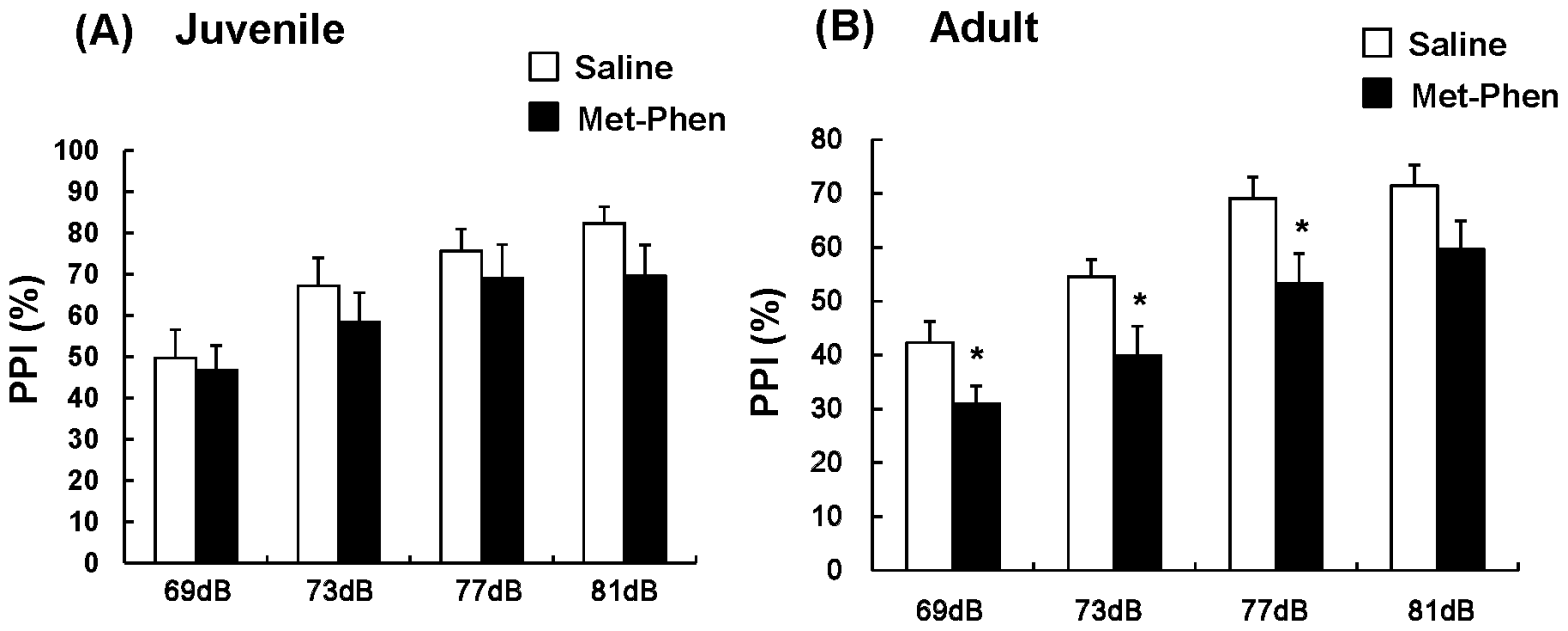
Auditory sensory gating PPI deficits after neonatal Met-Phen treatment. Saline (1.0 ml/kg/day) or Met-Phen (3.0 mg/kg/day) was administered i.p. from P7 to P9. Auditory sensory gating PPI test was performed at juvenile (5–6 weeks old) and adult stages (10–12 weeks old). Data represent the mean ± S.E.M. (n = 11 mice for control group, n = 12 or 13 for Met-Phen group). *P<0.05 compared with saline treated group.

Deficits in sensorimotor gating are a well characterized endophenotype of schizophrenia. **Figure 3** shows PPI data for juvenile and adult mice. MANOVA analysis of all data from test and control juvenile mice revealed no differences, indicating that treatment with Met-Phen (3.0 mg/kg/day for 3 days from P7–P9) did not cause PPI deficits in juvenile animals (**Figure 3A**). In contrast, MANOVA analysis of adult data revealed significant differences. Subsequent analysis showed that treatment with Met-Phen (3.0 mg/kg/day for 3 days from P7–P9) caused significant PPI deficits in three groups (69, 73, and 77 dB) (**Figure 3B**). No differences in startle response were found between the groups (data not shown).

### Effects of D-serine on PPI Deficits in Adult Mice after Neonatal Met-Phen Exposure

**Figure 4:**
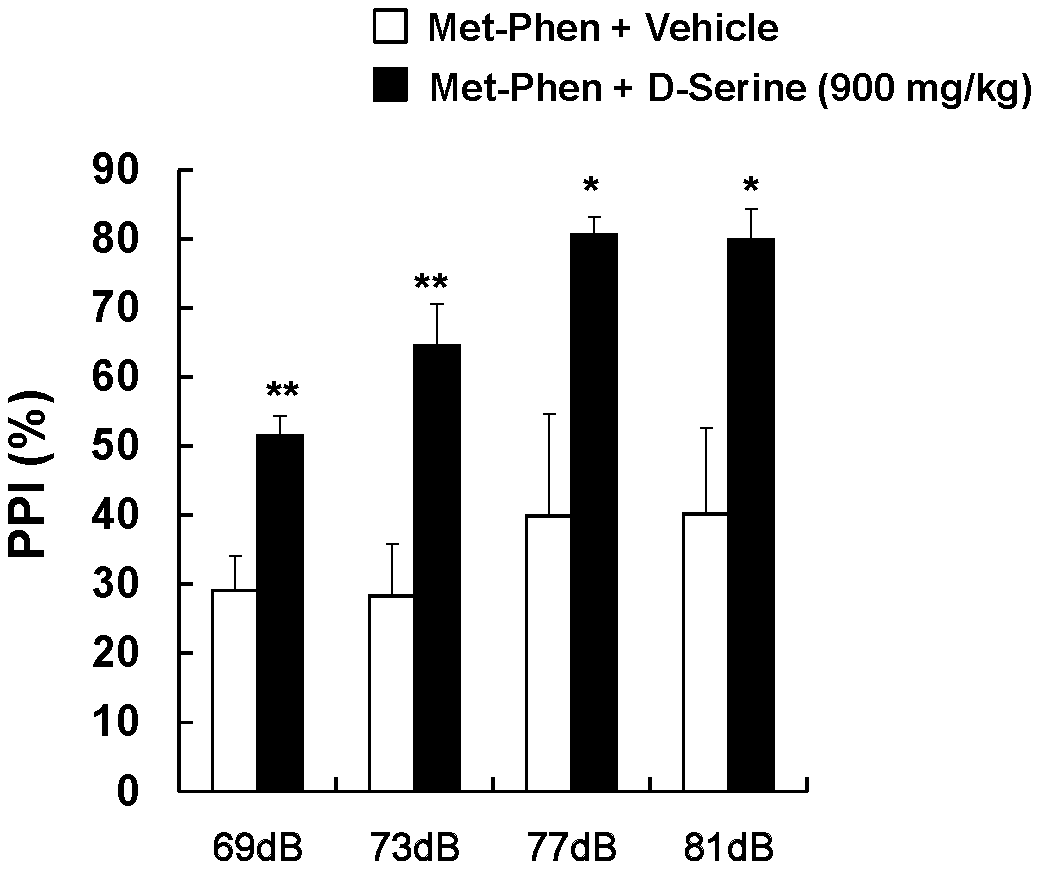
Effects of D-serine on PPI deficits at adult after neonatal Met-Phen treatment. Met-Phen (3.0 mg/kg/day) was administered i.p. from P7 to P9. PPI test was performed at adult (10–12 weeks old). D-Serine (900 mg/kg, i.p.) or vehicle (saline; 10 ml/kg) was administered 30 minutes before PPI test. Data represent the mean ± S.E.M. (n = 7 mice for control group, n = 12 for D-serine group). *P<0.05, **P<0.01 compared with Met-Phen+vehicle treated group.

We examined the effects of D-serine on PPI deficits in adult mice after neonatal Met-Phen exposure. D-Serine (900 mg/kg) or a vehicle was administered i.p., 30 minutes before PPI testing. MANOVA analysis revealed a significant effect for D-serine. Additional analysis showed that a single dose of D-serine significantly improved neonatal Met-Phen induced PPI deficits in all tested groups (69, 73, 77, and 81 dB) (**Figure 4**).

### D-serine Prevents PPI Deficits in Adult Mice after Neonatal Met-Phen Exposure

**Figure 5:**
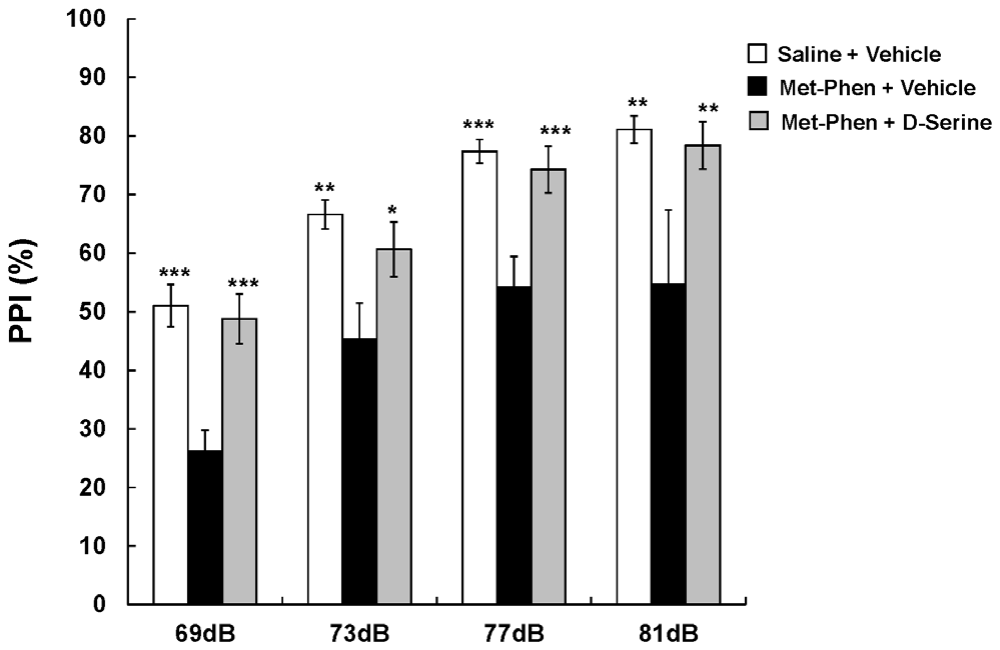
Preventive effects of D-serine on PPI deficits at adult after neonatal Met-Phen treatment. Met-Phen (3.0 mg/kg/day) was administered i.p. from P7 to P9. PPI test was performed at adult (11 weeks old). D-Serine (900 mg/kg/day, i.p.) or vehicle (saline; 10 ml/kg/day, i.p.) was administered chronically from P35 (5 weeks old) to P70 (10 weeks old). PPI test was performed 1 week (P77) after the final administration of D-serine. Data represent the mean ± S.E.M. (n = 5 mice for control group, n = 5 for D-serine group). *P<0.05, **P<0.01, ***P<0.001 compared with Met-Phen+vehicle treated group.

We examined whether D-serine was capable of preventing schizophrenia-like behavioral abnormalities in adult mice, after neonatal Met-Phen exposure. From P35 to P70, D-serine (900 mg/kg/day) or a vehicle (10 ml/kg/day) was chronically administered to mice with neonatal Met-Phen exposure. To exclude the acute effects of D-serine, PPI testing was performed one week after the last dose of D-serine or vehicle. As with clozapine, MANOVA analysis revealed a significant effect for D-serine. Subsequent analysis confirmed that chronic administration of D-serine significantly improved PPI deficits seen in neonatal Met-Phen exposure, in all the tested groups (69, 73, 77, and 81 dB) (**Figure 5**).

## Discussion

In this study, we found that neonatal administration of Met-Phen, an SSR inhibitor, caused behavioral abnormalities, such as increased rearing, and cognitive impairment in juvenile mice and cognitive impairment, and auditory sensory gating PPI deficits in adult mice. Furthermore, PPI deficits in adult animals with neonatal Met-Phen exposure were improved by a single dose of D-serine, indicating that D-serine could possess antipsychotic activity in this model. In this study, we found that the absolute values of PPI differed slightly between experiments (**Figures 3** **,** **4** **,** and **5**), suggesting a degree of variance between experiments. Moreover, we observed increased rearing activity in juvenile mice after neonatal Met-Phen exposure. This behavior may be due to alterations in amino acids levels within the cerebellum, since NMDA receptors in this region play a role in motor function. Considering the crucial role of D-serine in brain development, it is likely that disruption of NMDA receptor neurotransmission, by decreased D-serine levels during this period, may contribute to the later life behavioral abnormalities seen after neonatal Met-Phen exposure. Further detailed studies on how postnatal Met-Phen treatment induces schizophrenia-like behavioral abnormalities in adulthood are needed.

At birth, substantial amounts of D-serine were observed in all brain regions, including the cerebellum [16]. Levels of D-serine in the forebrain increased dramatically up to P21, and then remained roughly constant, whereas in the cerebellum, D-serine increased up to P7 and then declined dramatically to trace levels between P12 to P18 [16]. In this study, Met-Phen was administered from P7 to P9, since this period showed high levels of D-serine and SSR in all brain regions, including the forebrain and cerebellum [16]. Within 24 hours of the last dose of Met-Phen, levels of D-serine in mouse brains were significantly lower than those of the control group, indicating that Met-Phen inhibited SRR activity in the brain. Furthermore, there were alternations in the levels of glutamine and glutamate in the brains of Met-Phen treated mice, suggesting disruption of the glutamine-glutamate cycle in these animals. In the juvenile stage, Met-Phen treated mice showed increased levels of L-and D-serine in the cerebellum compared with controls. In adults, all amino acids were significantly lower in the hippocampus of Met-Phen treated mice relative to control mice, although no changes were observed in the frontal cortex and striatum. It is likely, that disrupting D-serine synthesis during development contributes to long-lasting changes in the synthesis and metabolism of amino acids associated with NMDA receptor function in the brain, in later life. These Met-Phen induced alterations to NMDA receptor neurotransmission persist through adulthood, even though Met-Phen is washed out from the adult brain.

In the NORT, the exploratory preference (14.2% in juveniles and 27.5% in adults) in mice after neonatal Met-Phen exposure was significantly lower than that of control mice, suggesting that the behavior of Met-Phen-treated mice may not be due to memory impairment. Reports show that repeated administration of phencyclidine (PCP) causes social interaction deficits in animals [47], [48]. In addition, negative symptoms such as social withdrawal are related to cognitive deficits in patients with schizophrenia [49]. Considering these findings, it is likely that our model of Met-Phen-treated cognitive deficits using the NORT, may show negative symptoms such as social withdrawal, which as stated before, are related to cognitive deficits [50]. Interestingly, we found that in the PCP-induced NORT model, D-serine could attenuate PCP-induced cognitive deficits in mice [51].

PPI deficits are generally recognized as an animal model of schizophrenia [52], as these deficits are observed in patients with psychiatric diseases, including schizophrenia [52]. In this study, we found that neonatal Met-Phen administration causes PPI deficits in adult, but not juvenile stages, suggestive of maturation-dependent auditory sensory gating deficits. In addition, adult mice with neonatal Met-Phen exposure, showed behavioral abnormalities, such as cognitive impairment, social interaction deficits and sensory gating PPI deficits, all of which are relevant to schizophrenia. We were able to improve these PPI deficits in adult mice with a single dose of D-serine. From the perspective of NMDA receptor related, neurodevelopmental processes playing a critical role in both normal brain development and schizophrenia, it is plausible that adult mice neonatally exposed to Met-Phen, may constitute an animal model of schizophrenia. A further detailed study will obviously be needed to confirm this hypothesis.

Accumulating evidence suggests that patients with schizophrenia show nonpsychotic and nonspecific prodromal symptoms such as depression, social withdrawal, and cognitive impairment, for several years preceding the onset of frank psychosis [53]–[58]. A recent meta-analysis of 27 studies showed that the average rate of transition to full psychosis among such patients is 22% within the first year and 36% within three years [57]. Therefore, providing early intervention at the prodromal phase of schizophrenia and related psychosis is one of the most important and challenging tasks in psychiatry [58]. In this study, we found that neonatal Met-Phen treatment induced behavioral abnormalities, such as stereotypy, cognitive impairment and social withdrawal in juveniles, suggesting that these mice may show prodromal, or at risk of psychosis symptoms. Interestingly, we found that chronic administration of D-serine from pre-adolescent to adult stages prevented the onset of PPI deficits in adult mice. A recent report on two pilot studies using prodromal subjects showed that glycine, an endogenous agonist of the NMDA receptor, reduced symptoms with promising effect sizes [59]. This would imply that targeting the glycine agonist sites of the NMDA receptor could provide promising therapy for the prodromal phase of psychotic disorders. This makes D-serine an attractive potential drug for early intervention in the onset of schizophrenia, since D-serine is effective in treating several symptoms of schizophrenia [25]–[27], [60].

Before D-serine could be considered as a therapeutic agent, it would be necessary to solve the problem of bioavailability. Orally administered D-serine is heavily metabolized by D-amino acid oxidase (DAAO) in peripheral organs, diminishing its bioavailability in human subjects. Administering higher doses could potentially lead to nephrotoxicity, although no significant adverse events have as yet been observed at doses of up to 4 g/day [19]. Therapeutic levels of D-serine at lower doses could be achieved if D-serine were co-administered with DAAO inhibitors [17], [46]. This combination could represent a new pharmacological intervention for the onset of schizophrenia and related psychosis.

Finally, as with studies of this nature, there are some limitations to this report. The main limitation was the use of Met-Phen, particularly since the precise pharmacology of Met-Phen as an SRR inhibitor has not been fully elucidated. Although several SSR inhibitors have been reported, there is no data on the selectivity of these inhibitors [9]. Therefore, further studies using selective SSR inhibitors or SSR gene deletion during the neonatal period are needed. It is also reported that phenazine (Phen) is an inactive inhibitor at SSR [36]. Thus, these experiments could be strengthened if Phen were used as a negative control for this study. Another limitation was the noted changes on brain amino acid levels, after neonatal Met-Phen exposure. In this study, we found alternations in brain levels of other amino acids including glutamate, glutamine, glycine and L-serine, all of which could affect NMDA receptor neurotransmission [5], [38], [39], [61], [62]. It is reported that the glutamine-glutamate cycle and L-serine-glycine pathway, play a role in NMDA receptor neurotransmission in the brain [5], [39], [61], [62]. Given the key role of the NMDA receptor in the pathophysiology of schizophrenia [1]–[6], [63], our model represents a new tool for evaluating the contribution of NMDA receptor hypofunction in the pathophysiology of schizophrenia. Accumulating evidence suggests that the potent antioxidant glutathione plays a role in the modulation of redox-sensitive sites on the NMDA receptor and in the pathophysiology of schizophrenia [64]–[70]. Since glutathione is synthesized in the body from L-glutamate, glycine and L-cysteine, it would also be of great interest to measure the brain glutathione levels of animals in this model.

In conclusion, our results suggest that neonatal Met-Phen exposure causes behavioral abnormalities, relevant to prodromal symptoms and schizophrenia during juvenile and adult stages, respectively. Interestingly, chronic administration of D-serine from juvenile to adult stages may prevent the onset of schizophrenia-like symptoms in adults after neonatal Met-Phen exposure, indicating that D-serine may serve as an early intervention for psychosis. It is therefore likely that neonatal Met-Phen treatment may constitute a new neurodevelopmental animal model of schizophrenia, based on the NMDA receptor hypofunction hypothesis.

## Supporting Information

Table S1 **Effect of neonatal Met-Phen treatment on levels of amino acids in the brain at juvenile.**
(XLSX)

Table S2 **Effect of neonatal Met-Phen treatment on levels of amino acids in the brain at adult.**
(XLSX)
